# Differential Effects of Conservational Management on SOC Accumulation in the Grasslands of China

**DOI:** 10.1371/journal.pone.0137280

**Published:** 2015-09-10

**Authors:** Ping Zhang, Jie Tang, Wenjuan Sun, Yongqiang Yu, Wen Zhang

**Affiliations:** 1 Key Laboratory of Groundwater Resources and Environment, Ministry of Education, Jilin University, Changchun, 130021, P.R. China; 2 LAPC, Institute of Atmospheric Physics, Chinese Academy of Science, Beijing, 100029, P.R. China; 3 LVEC, Institute of Botany, Chinese Academy of Science, Beijing, 100093, P.R. China; Institute for Sustainable Plant Protection, C.N.R., ITALY

## Abstract

Conservational management practices in grasslands have been considered one of the efficient options to enhance the soil organic carbon (SOC) accumulation. However, the SOC changes after the conservational management practices vary significantly under different grassland vegetation types and the environmental conditions. At present, it is not clear how the SOC accumulation changes along the soil profile if conservational management practice was adopted. In this study, we collected 663 paired observational data of SOC changes with and without conservational management practices in grasslands of China from 176 published literatures that has both the surface (0‒20 cm) and subsurface (to 40 cm depth) SOC measurements. The differences of SOC density (SOCD) between pre‒management and post‒management in the vertical soil layers were analyzed in order to establish a quantitative relationship of the SOC changes between the subsurface and the surface. The results revealed that in all grasslands, conservational management practices benefits the SOC accumulation by enhancing 0.43‒1.14 Mg C ha^–1^ yr^–1^. But the SOC increment weakened downwards along the soil profile. While the surface SOC was enhanced by 17% after conservational management, the subsurface SOC was enhanced by only 7%. The SOC accumulation was closely correlated with restoration duration, pre-management SOCD and the environmental factors and differed greatly among different grasslands and the practices adopted. The alpine and mountain grassland showed a higher annual SOC increment than the temperate grassland with the annual rate of 1.62 and 0.72 Mg C ha^-1^ yr^-1^, respectively. The SOC increment caused by the artificial plantation and the grazing exclusion conservational management was more than 2-fold that of the cropland abandonment and the extensive utilization. With the quantitative relationship of the SOC changes between soil layers, we provide a methodological option to estimate SOC changes to layers deeper than the recommendation of IPCC when only the surface layer SOC increment is available.

## Introduction

Soil carbon is the most important reservoir of terrestrial carbon [1, 2], and 2‒4 times more carbon is stored in soil compared with aboveground biomass [3]. In recent decades, soil organic carbon (SOC) decomposition has accelerated, and soil CO_2_ emissions have increased because of more intensive land use [4]. However, SOC has a longer residence time and lower decomposition rate compared with fossil fuel combustion and can act as a carbon sinks when conservation management practices were implemented [5, 6]. Consequently, soil is an important natural carbon sink for greenhouse-gases released by fossil fuel combustion and land-use changes [7–11]. Grasslands are an important component of terrestrial ecosystems and, exhibit a strong carbon sequestration potential [12]. Carbon accumulation in grassland ecosystems occurs mostly below ground [6], and it may be modified by land-use change [8, 13].

From the late 1980s and early 2000s, almost 90% of the grassland in China was over-exploited for cultivation and grazing in an attempt to feed the increasing population, consequently, the decomposition of SOC increased [14]. To impede grassland retrogression, the ‘Grain for Green Program’ was implemented in 2000 in arid and semi-arid areas of China [15]. A suite of recommended management practices for improving soil C storage in grassland ecosystems, such as cropland abandonment, grazing exclusion, soil fertilization, sustainable grazing, and artificial planting, were employed [7, 13, 16–18]. The increase in SOC from conservation management can offset the carbon emissions caused by poor management and fossil fuel combustion [19, 20]. An estimation of the amount of carbon sequestered by conservational management practices requires information on carbon accumulation by various vegetation types and the management activities [21]. Site-scale experiments and measurements have improved our knowledge of the laws and underlying mechanisms of carbon dynamics. However, the labor involved in soil sampling and limited numbers of samples collected from subsurface layers has restricted the assessment of carbon stocks variation, especially at large scales [22, 23].

At present, most of the estimations of SOC accumulation are inferred from surface SOC, whereas the data from deeper soil layers are limited [24–27]. A shallow sampling may underestimate the total SOC sequestration under conservational management if SOC changes along a soil profile are not accounted for [28]. Estimations of changes in SOC at deeper soil horizons must be considered especially because these changes are responsive to disturbances at the soil surface [29–31]. Subsoil soil carbon can accumulate through the transportation of surface layer SOC and decomposition of roots and soil organic matter [27, 32, 33]. Therefore, the vertical profile of soil carbon can be estimated by using surface SOC observations, depending on the parameterized relationship of SOC between the surface and subsurface layers [34]. The SOC profiles generally result from an addition of legacy SOC distributions and a vertical distribution of root’s deposits among different grass species [35, 36]. In addition, the amount of carbon sequestered depends on factors that include the initial SOC content, land-use legacy, and climatic conditions in the ecological area [9, 37‒40].

Since 2000, the Chinese government has promoted a suite of projects to restore degraded and malfunctioning grasslands and protect rangeland resources [41]. A large area of grassland was managed by recommended practices to prevent degradation. In addition, numerous experimental studies were conducted to monitor SOC dynamics in response to conservational management in grasslands. These studies covered most regions of temperate, mountain, and alpine grasslands. Analyzing the information in previously published studies is an effective approach to improving our knowledge on how changes in management affect SOC stocks [9, 11]. We aimed to characterize the determinants of surface-layer SOC dynamics and calculate the vertical distribution of SOC accumulation using published literature (S1 Table).

## Material and Methods

### Data collection and processing

**Fig 1:**
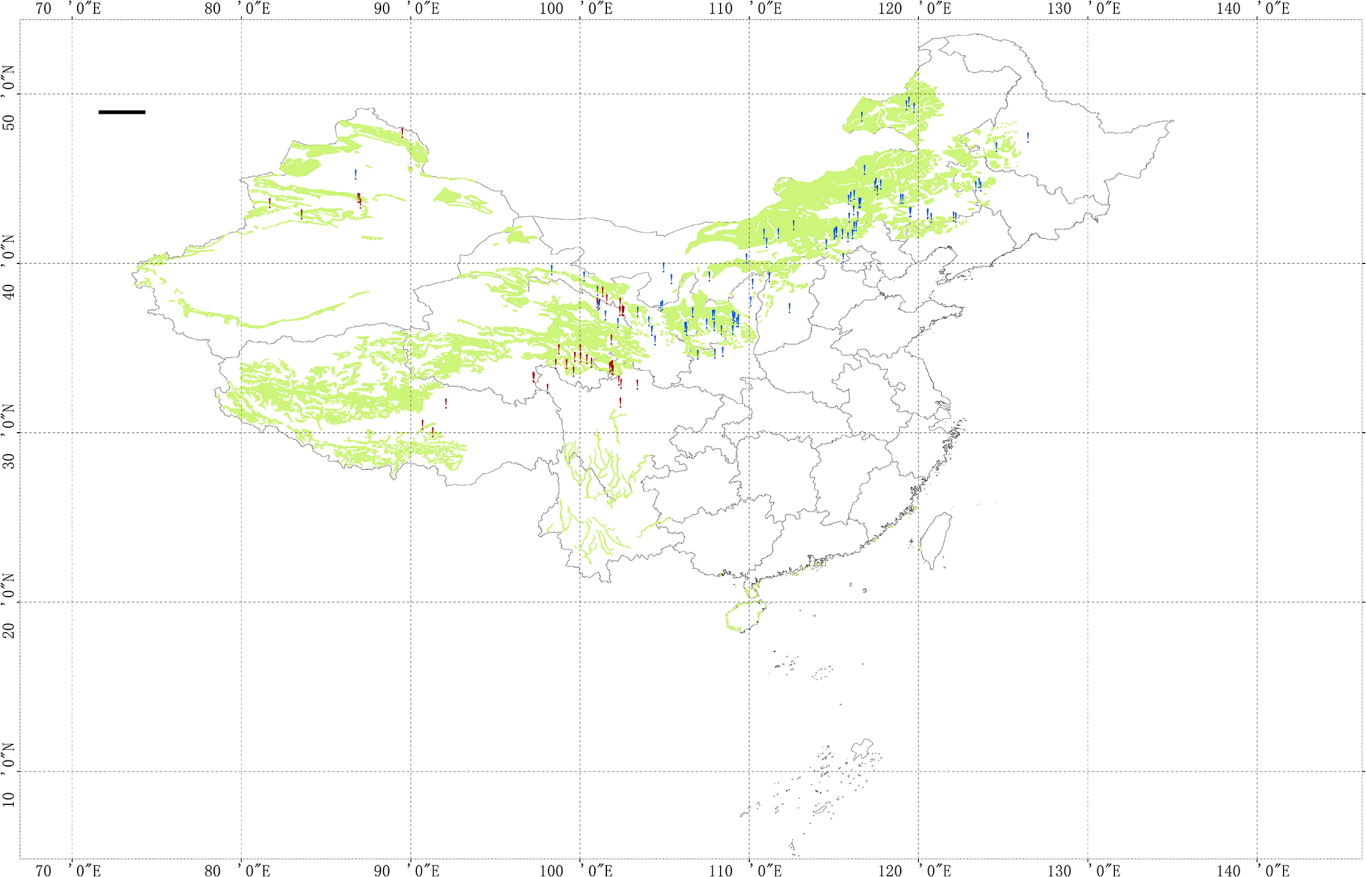
Location of the 131 sites used in the present study. The green area refers to grassland area. The red dot indicates the alpine grassland + mountain grassland, and the blue dot indicates temperate grassland. (The data set is provided by Data Center for Resources and Environmental Sciences, Chinese Academy of Science (RESDC)).

We collected data of SOC changes in grasslands by searching literatures using the Institute for Scientific Information (ISI) Web of Science and China National Knowledge Infrastructure (CNKI). We only retrieved original data from field measurements conducted at depth of 0‒20 cm and 0‒30 cm in Chinese grasslands. Measurements of the SOC changes over soil depths of 0‒40 cm that were reported in certain studies were included in the database. Only studies that determined SOC (% or g kg^‒1^) using the Walkley-Black wet oxidation method were included, and those without complete information on the soil depth or pre-management SOC measurements were excluded. The final version of the database used in our analysis included 663 paired observations from 176 publications, of which 386 paired data points were used to document the restoration information over a time period ranging from 1 to 100 years (S1 Table). The spatial distribution of the selected sites distributed across the major grassland ecosystems in China is shown in Fig 1. The geographic information (longitude, latitude and altitude) and meteorological information (mean annual precipitation and mean annual temperature) were retrieved directly from published literature and closely related publications.

The SOC differences between the managed and unmanaged grasslands were calculated using paired field measurements. Two methods were used to derive the paired measurements: for the measurements obtained from direct-paired experiments, the intact plot and neighboring restoration plot acted as the control (unmanaged) and managed plots, respectively; and for chronological measurements at a single site, the SOC prior to and after management acted as the control and managed plots, respectively. The grasslands of the study area were categorized into the following two groups according to the dominant grass species, a temperate grassland (TG) and an alpine grassland +mountain grassland (AG+MG). The TG consists of steppe and meadow grassland in the northern part of China, including Inner Mongolia, Xinjiang, Gansu, Heilongjiang and Ningxia provinces. Previous studies have shown that AG+MG have similar SOC [42, 43]; therefore, they were merged into one group. The TG group consisted of 91 sites, and the AG+MG group consisted of 40 sites (Fig 1). Four conservational management practices were identified in the study: extensive utilization (EU), grazing exclusion (GE), artificial plantation (AP) and cropland abandonment (CA). EU refers to fields that were subjected to recommended grazing practices; GE refers to fields in which grazing was excluded inside a fenced area, with free grazing outside the fenced area; AP refers to fields that were sown with vegetation, legumes, and pea-shrubs; and CA refers to fields that were converted from abandoned cropland to natural grassland. During the entire fallow stage, only the abandoned cropland was naturally restored.

### Calculation of SOC density

The SOC density (SOCD, Mg C ha^-1^) in a given horizon was calculated using Eq 1

$$SOCD=SOC\times BD\times H\times 10^{-1}$$ (1)

where SOC and BD are the SOC concentration (g kg^-1^) and bulk density (g cm^-3^), respectively; and *H* is the horizon thickness (cm). A simple conversion equation (SOC = 0.58×SOM) was used when only the soil organic matter (SOM) was available in the literature. The constant 0.58 is the Bemmelen index, which is used to convert organic matter concentration to organic carbon content [39]. If the BD was not available in the literature, the value was estimated from negative exponential equations, and the statistical significance of the referenced equation was shown in Table 1.

**Table 1:** Agreement between the measured and the calculated BD with the referenced equations.

Land use type	BD equation	Reference	N	R^2^ †
TG	*BD* = 1.6085×*e* ^(−0.01244×*SOC*)^	[42]	311	0.433**
AG+MG	*BD* = 0.3+1.28×*e* ^(−0.0172×*SOC*)^	[44]	80	0.469**
CA	*BD* = 1.3770×*e* ^(−0.0048×*SOC*)^	[45]	19	NS

†indicated the significance the referenced regression equation.

**, significance at <0.01 level. SOC (g kg^-1^)

### Statistical analysis

We used ΔSOCD to represent the difference between pre- and post-management SOCD. The ΔSOCD of the 0‒20 cm, 20‒30 cm and 30‒40 cm soil horizons were calculated and statistically analyzed using paired t-test. In addition, the relationship between the surface ΔSOCD (0‒20 cm depth) and sub-surface horizons ΔSOCD (20 cm and lower) was analyzed. A Pearson correlation analysis and partial correlation analysis were performed to determine the factors affecting ΔSOCD. Subsequently, regression equations were developed to estimate the ΔSOCD and post-management SOCD (SOCD_post) under various environmental conditions.

## Results

### SOCD and ΔSOCD in different grasslands

**Fig 2:**
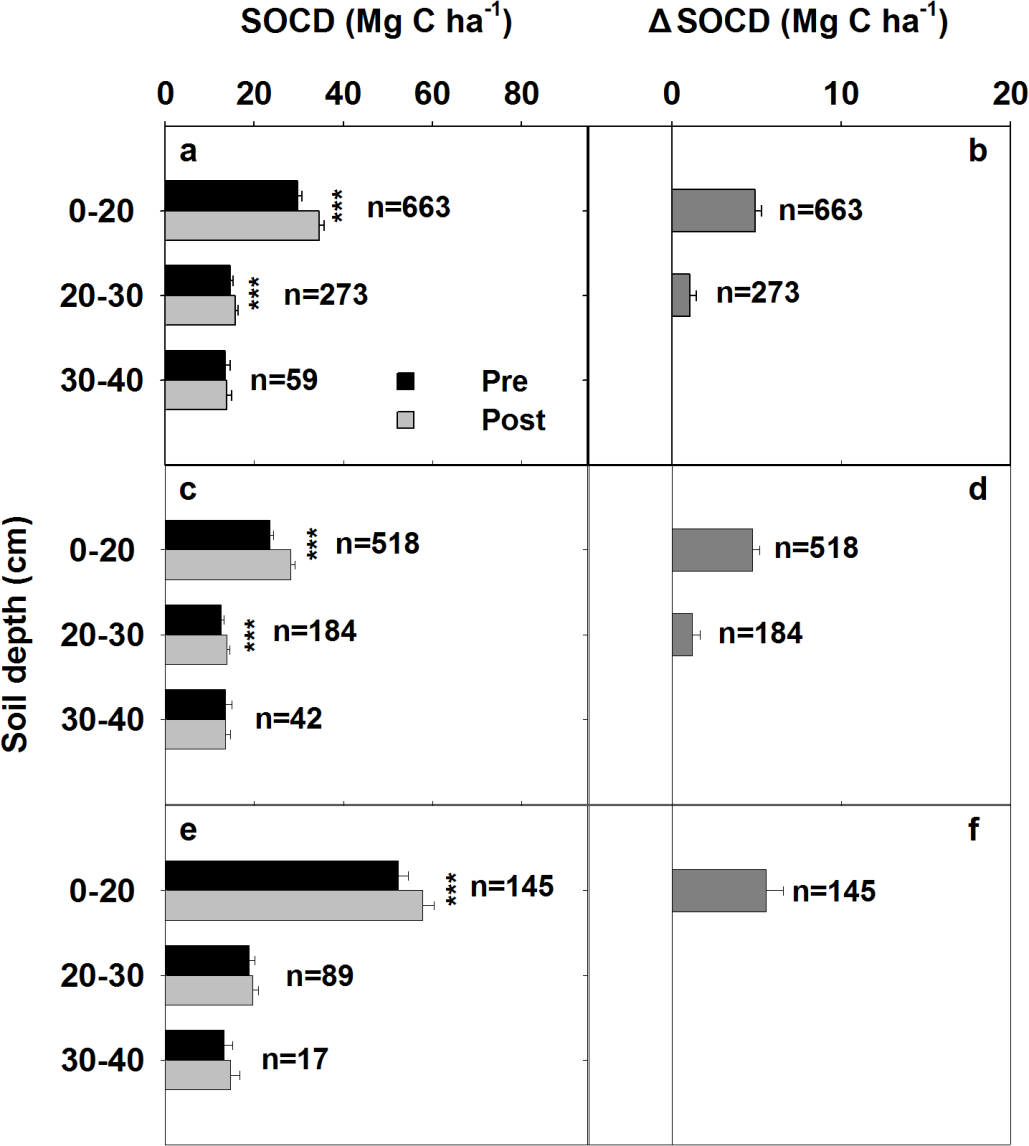
Profile of SOCD and ΔSOCD in 0‒40 cm soil layer: for all grasslands (a, b), temperate grasslands (c, d), and alpine grassland+mountain grassland (e, f). The error bar represents the standard error. **and * represent the significance of the t-test at the 0.01 and 0.05 levels, respectively.

An analysis of all of the paired SOCD data, indicated increases of 17% and 7% at depth of 0‒20 cm and 20‒30 cm depths, respectively, in the grassland following conservational management (Fig 2A and 2B). The SOCD and ΔSOCD differed substantially between different grassland types. The SOCD was lower in TG than in AG+MG for all horizons (Fig 2C and 2E). However, the SOCD in TG exhibited a greater increment, with 20% and 10% increases at depth of 0–20 cm and 20‒30 cm, respectively (Fig 2D). In AG+MG, however, the SOCD at 0‒20 cm increased by 11% (Fig 2F) and changes in SOCD were not statistically significant at deeper layers (Fig 2E and 2F). However, AG+MG had a higher annual ΔSOCD compared with that of TG (1.62 vs 0.72 Mg C ha^-1^ yr^-1^; Table 2).

**Table 2:** Annual carbon sequestration rate in the 0‒20 cm soil horizon in different grasslands and under different management practices.

Groups	Subgroups	Annual carbon sequestration rate (Mg C ha^-1^ yr^-1^)	ANOVA	Number of observations
Vegetation	TG*	0.72±0.10†	b	338
	AG+MG	1.62±0.39	a	48
Managements	AP	1.14±0.29	a	82
	CA	0.43±0.11	b	81
	EU	0.49±0.26	b	72
	GE	1.04±0.16	a	151

*, TG and AG+MG indicate temperate grassland and alpine grassland+mountain grassland, respectively. AP, CA, EU and GE indicate artificial plantation, cropland abandonment, extensive utilization and grazing exclusion, respectively.

†, standard error

### SOCD and ΔSOCD under different conservational management practices

**Fig 3:**
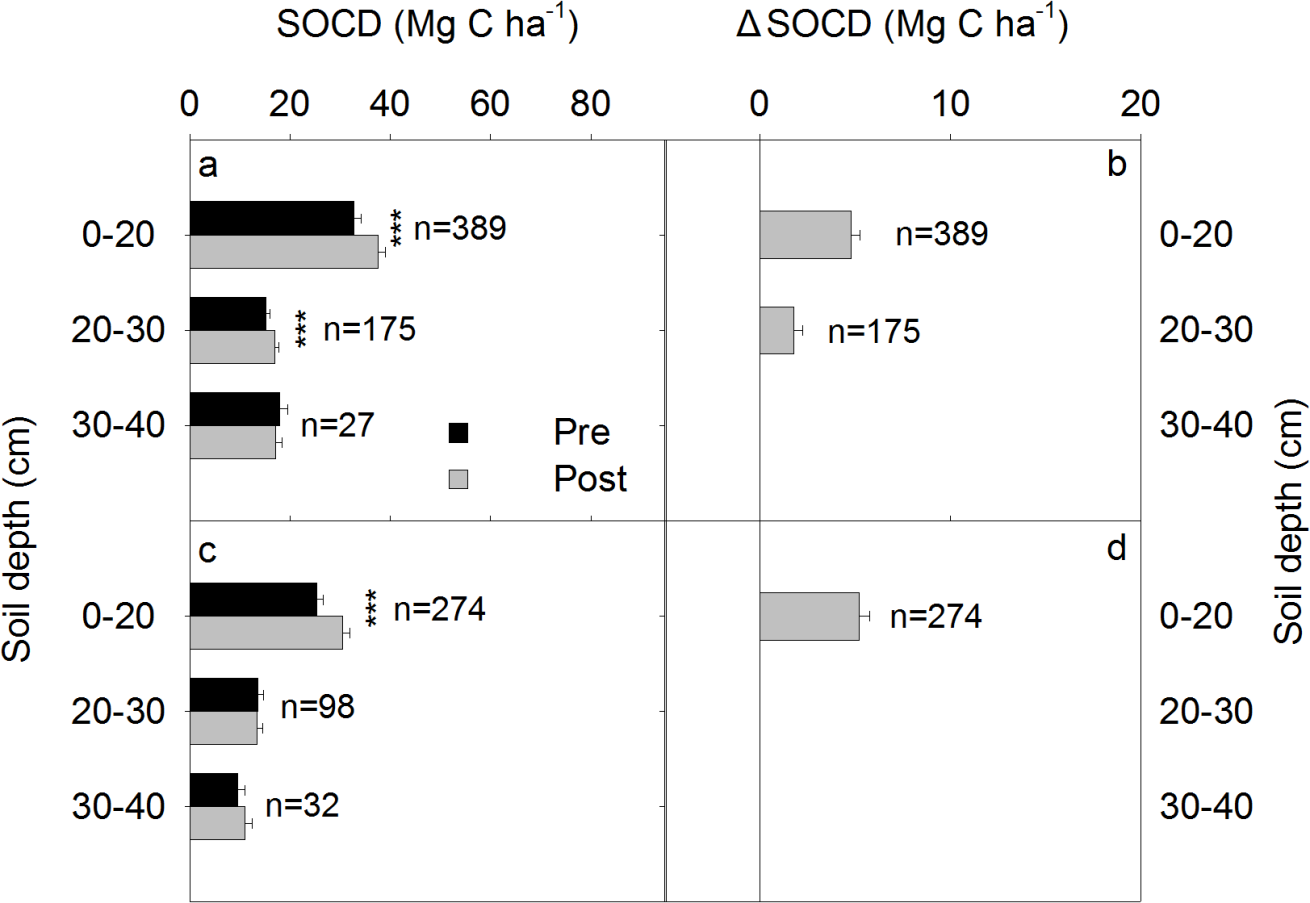
Profile distribution of SOCD and ΔSOCD in the 0‒40 cm layer of the soil for extensive utilization + cropland abandonment (a, b) and grazing exclusion + artificial plantation (c, d). The error bar represents the standard error. **and * represent the significance of the t-test for the paired sample at the 0.01 and 0.05 levels, respectively.

The AP and GE exhibited a similar annual ΔSOCD value, which were significantly higher than that of the CA and EU (Table 2), which also exhibited similar annual ΔSOCD values (Fig 3). The SOCD at 0‒20 cm and 20‒30 cm increased by 15% and 21%, respectively, in response to AP and GE management (Fig 3B). However, under CA and EU, only the SOCD at a depth of 0‒20 cm increased by 21% and there was no increase in SOCD in the deeper soil layers.

### Relationship between ΔSOCD at different soil depths

**Fig 4:**
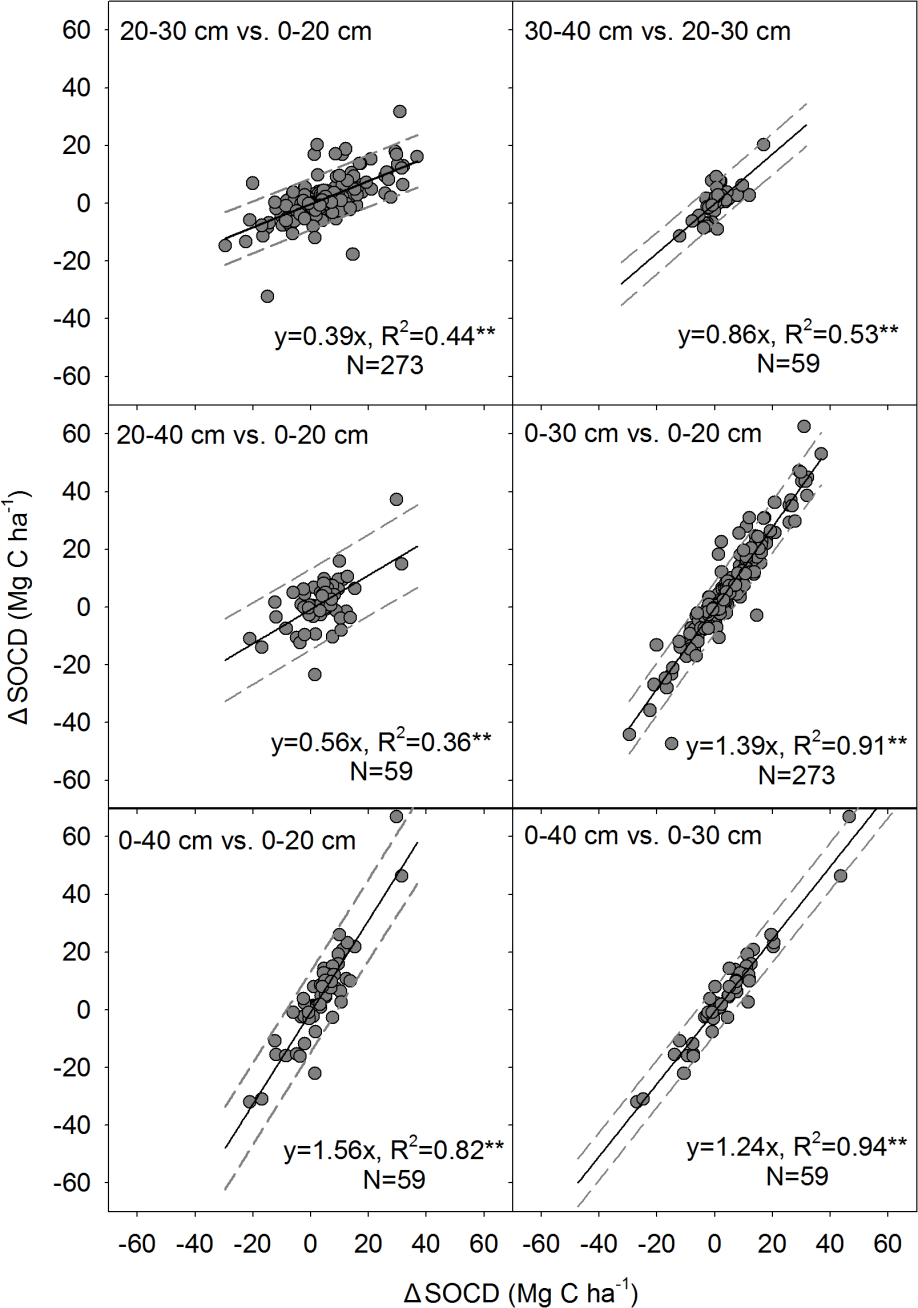
The linear relationship between ΔSOCDs in different soil layers. The dashed lines represent the 95% confidence intervals. The y-axis represents the deeper layers, and the x-axis represents the upper soil layers.

Significant linear relationships between the ΔSOCDs at different soil depths were observed (Fig 4). The linear relationship was in the form *y* = k×*x*, where *y* and *x* represent the ΔSOCD at different soil depths and, k represents a quantitative parameter of the relationship. The SOCD increased 0.42‒0.45 Mg C ha^-1^ yr^-1^ at depth of 20‒30 cm and 20‒40 cm in response to CA and EU when the surface soil layer (0‒20 cm) SOCD increment reached 1 Mg C ha^-1^ yr^-1^ (Table 3). Similarly, the SOCD at depths of 20‒30 cm and 20‒40 cm increased by 0.26 and 0.67 Mg C ha^-1^ yr^-1^, respectively, in response to AP and GE when the surface soil layer SOCD increment reached 1 Mg C ha^-1^ yr^-1^. In general, the SOCD increments in the 0‒30cm and 0‒40 cm soil layers were 1.24 and 1.65-fold higher, respectively than that of 0‒20 cm soil layer (Fig 4).

**Table 3:** The linear parametric relationship between ΔSOCD (Mg C ha^-1^), ΔSOCD/10 cm (Mg C per 1000 m^3^) at different soil depths*.

	Depth (cm)	ΔSOCD				ΔSOCD/10 cm			
		k	R^2^	n	Sig.	k	R^2^	n	Sig.
CA+EU	20‒30 vs 0‒20	0.45	0.56	175	<0.001	0.45	0.522	175	<0.001
	20‒40 vs 0‒20	0.42	0.26	27	<0.01	0.42	0.26	27	<0.01
	0‒30 vs 0‒20	1.45	0.93	175	<0.001	0.96	0.92	175	<0.001
	0‒40 vs 0‒20	1.42	0.80	27	<0.001	0.73	0.81	27	<0.001
	0‒40 vs 0‒30	1.17	0.94	27	<0.001	0.88	0.93	27	<0.001
AP+GE	20‒30 vs 0‒20	0.26	0.21	98	<0.001	0.53	0.21	98	<0.001
	20‒40 vs 0‒20	0.67	0.45	32	<0.001	0.67	0.41	32	<0.001
	0‒30 vs 0‒20	1.26	0.86	98	<0.001	0.84	0.86	98	<0.001
	0‒40 vs 0‒20	1.67	0.83	32	<0.001	0.83	0.80	32	<0.001
	0‒40 vs 0‒30	1.29	0.94	32	<0.001	0.97	0.93	32	<0.001

*, ΔSOCD (Mg C ha^-1^ yr^-1^) is the change in SOCD (Mg C ha^-1^ yr^-1^) caused by grassland management with respect to unmanaged grasslands. ΔSOCD/10 cm (Mg C per 1000 m^-3^) is the change in SOCD per volume (1000 m^3^)

### The factors affecting ΔSOCD in the surface soil layer

The surface (0‒20 cm) layer ΔSOCD was positively correlated with the restoration duration (RD), altitude above sea level (ALT) and geographic latitude (LAT), and negatively correlated with the SOCD before management (SOCD_pre), mean annual temperature (MAT), mean annual precipitation (MAP) and geographic longitude (LONG) (Table 4). The RD appeared to dominate the ΔSOCD, although other environmental factors also regulated the SOCD.

**Table 4:** Pearson correlation analysis and partial correlation analysis between eight variables.†

		ΔSOCD	SOCD_pre	RD	MAP	MAT	ALT	LAT	LONG
Pearson	ΔSOCD	1							
	SOCD_pre	−0.053	1						
	RD	0.347**	−0.171**	1					
	MAP	−0.058	−0.116**	0.041	1				
	MAT	−0.092	−0.482*	0.302**	−0.06	1			
	ALT	0.067	0.365**	−0.057	0.235**	−0.374**	1		
	LAT	0.022	0.034	−0.181**	−0.336**	−0.384**	−0.660*	1	
	LONG	−0.093	−0.128*	−0.062	0.016	−0.053	−0.694**	0.665**	1
Partial	ΔSOCD	1							
	SOCD_pre	−0.134*	1						
	RD	0.399**	0.021	1					
	MAP	−0.071	−0.240**	0.052	1				
	MAT	−0.090	−0.198**	0.118*	−0.426**	1			
	ALT	0.016	0.073	−0.010	−0.252**	−0.867**	1		
	LAT	0.031	−0.048	−0.030	−0.502**	−0.884**	−0.860**	1	
	LONG	−0.086	0.065	0.024	0.296**	−0.142**	−0.345**	−0.066	1

Notes: ΔSOCD (change in SOCD between post and pre-management, Mg C ha^-1^), SOCD_pre (pre-management SOCD, Mg C ha^-1^), RD (restoration duration, yr), MAP (mean annual precipitation, mm), MAT (mean annual temperature, °C), ALT (altitude above sea level, m), LAT (geographic latitude, °) and LONG (geographic longitude, °) are the variables.

* significance at the <0.05 level;

** significance at the <0.01 level.

A pre-analysis showed that the factors affecting the ΔSOCD in the grasslands differed between the regions that were north and south of 40°N altitude. A stepwise regression was performed for grasslands in the two regions, and regression equations were developed (Table 5). In the LAT<40° area where AG+MG dominates, the ΔSOCD increased with RD and decreased with geographic longitude. In the LAT>40° area where TG dominates, the ΔSOCD increased with RD, ALT and decreased with MAT and SOCD_pre. Under the same RD, there may be greater SOC accumulation in the LAT<40° area than in the LAT>40° area. The SOCD_post, which was the summation of SOCD_pre and ΔSOCD showed a similar regression equation with similar parameters (Table 5).

**Table 5:** Stepwise regression of ΔSOCD and SOCD_post in the 0‒20 cm soil layer.

	Equation	R^2^	N	Sig.
All	ΔSOCD = 22.36+0.267×RD–0.687×MAT–0.07×SOCD_pre–0.007×MAP–0.114×LONG	0.194	373	<0.001
LAT<40°N	ΔSOCD = 42.02+0.265×RD–0.386×LONG	0.264	187	<0.001
LAT>40°N	ΔSOCD = 3.73+0.213×RD –0.685×MAT –0.18×SOCD_pre+0.006×ALT	0.237	186	<0.001
All	SOCD_post = 22.36+0.267×RD–0.687×MAT+ 0.93×SOCD_pre–0.007×MAP–0.114×LONG	0.861	373	<0.001
LAT<40°N	SOCD_post = 44.02+0.265×RD –0.404×LONG +0.995×SOCD_pre	0.901	187	<0.001
LAT>40°N	SOCD_post = 3.73+0.213×RD –0.685×MAT +0.82×SOCD_pre+0.006×ALT	0.819	186	<0.001

Notes: ΔSOCD (change in SOCD between post and pre-management, Mg C ha^-1^), SOCD_pre (pre-management SOCD, Mg C ha^-1^), RD (restoration duration, yr, 1‒100), MAP (mean annual precipitation, mm, 80‒750), MAT (mean annual temperature, °C, −5‒11), ALT (altitude above sea level, m, 100‒4000), LAT (geographic latitude, °, 33‒50) and LONG (geographic longitude, °, 80‒125) are the variables.

## Discussion

### SOC accumulation by conservational management and the underlining mechanism

Conservational management resulted in carbon accumulation in the surface layer and the subsurface layer deep to a depth of 40 cm, which accounted for most of the SOC variation in the entire soil profile [46]. The conservative management practices, such as GE, CA, AP and EU, counteracted or alleviated disturbances caused by animals, which benefited the regeneration of grassland and accelerated the recovery of aboveground and belowground biomass [47]. The aboveground litter and belowground roots were the major carbon inputs to the SOC [48‒50], and the turnover of the litter and roots cascade carbon input to the grassland soils [6, 51]. Furthermore, the enhanced organic material input improved the stabilization of SOC in aggregates, which tended to be recalcitrant to decomposition [6, 52‒54]. In addition, grassland regeneration also altered the community species composition related to livestock diet selections [20, 55, 56], which eventually resulted in a fibrous rooting system and increased the SOM formation and accumulation [57]. Therefore, conservational management, such as CA can improve vegetation cover and mitigate potential exposure to wind and rain erosion [47, 58].

The SOC accumulation rate averaged across all conservation management practices was 0.83 and 1.30 Mg C ha^-1^ yr^-1^ for the 0‒20 cm and 0‒40 horizons, respectively, which closely approximates the SOC sequestration rate for GE in a study by Wang et al. (2011) [11]. The annual carbon sequestration for set-aside management practices (similar to fencing exclusion) under the US Conservation Reserve Program (CRP) was 1.20 Mg C ha^-1^ (0‒30 cm) [59]. In our study, the SOC accumulation rate in the 0–30 cm layer was 0.86 Mg C ha^-1^ yr^-1^, which was lower than that in the CRP application area. The managed TG in China had an annual SOC accumulation rate of 0.73 Mg C ha^-1^ yr^-1^ compared with that of the AG+MG which had an accumulation rate of 1.70 Mg C ha^-1^ yr^-1^. Therefore, the carbon sequestration potential of AG+MG exceeds that of North American grassland ecosystems.

SOCD_0‒40 cm_ values of approximately 57 Mg ha^-1^ have been reported for AG [43], whereas values of 49 Mg ha^-1^ have been reported for TG [42]. This result indicates that with the same initial SOC content, managed AG+MG have a greater SOC accumulation compared with TG., The aboveground biomass and belowground biomass, which are the most important carbon inputs for SOC, of the TG was approximately twice that of the AG [42, 44]. Higher annual SOC accumulation for the AG+MG may have been a result of the cold and wet climatic conditions (MAT = −1.3°C and MAP = 524 mm for the AG+MG, and MAT = 4.8°C and MAP = 386 mm for the TG), which benefitted SOC accumulation and reduced SOC decomposition [18, 60]. Furthermore, the differences in SOC accumulation between the grassland types may have been caused by the different components of soil aggregates, which could preclude the decomposition of SOC during formation [61]. The different SOC components, including the light fraction organic carbon (LFOC) and heavy fraction organic carbon (HFOC), varied with disturbance [62]. The HFOC is resistant to decomposition, and the LFOC undergoes mineralization [53, 62, 63]. The proportion of LFOC and HFOC in the TG, AG and MG remains unknown. The possible mechanisms underlying the formation and breakdown of soil aggregates and their effect on SOC accumulation should be explored in future work.

Most of the TG has undergone intensive land-use change since the 1980s [49], whereas the AG distributed across the Tibetan Plateau did not undergo significant land-use change through the 1980s and 2004 [44, 64]. The averaged RD was 6.7 and 12.6 years for the AG+MG and TG, respectively. During the initial period of grassland regeneration, sites are more productive and likely accumulate more soil C [65]. The AG+MG, which was in the early stage of regeneration, exhibited a higher SOC sequestration rate. In contrast, the TG was in the mid-stage of regeneration and showed a decelerated SOC sequestration rate.

### Effects of different management practices

Among the various management practices, AP and GE resulted in a higher carbon sequestration rate compared with CA and EU. Both AP and GE tended to protect the physical aggregates of SOC without disturbance. Wu et al. (2010) demonstrated that AP of *Elymus nutans* grassland in an alpine meadow decreased soil pH, and the acidic conditions were favorable for litter decomposition [66]. Most AP is intended to increase the aboveground biomass and further increase the SOC input [49]. GE with the application of fencing not only can increase biomass but reduce decomposition [18]. In the current study, AP and GE were characterized by extensive interference or no interference, respectively, and these conditions were advantageous to SOC accumulation. Conversely, CA and EU were affected by human disturbance, which mostly intensified SOC decomposition. Although CA was not disturbed during the regeneration period, its SOCD_pre was lower than that of the other restoration managements. The legacy of agriculture resulted in scarce nutrient availability and reduced soil seed banks, which restricted the community development of community [53, 67]. A naturally regenerated grassland on fallow cropland showed a lower carbon input than did the AP and GE, especially during the initial phase of regeneration [68]. Although Conant and Paustian (2002) demonstrated that a recommended grazing density was beneficial for SOC accumulation [69], the increased abundance of sedges at the expense of forbs reduced belowground production [20]. A mowing substitute for grazing decreased carbon input [11], and plowing and harrowing were partly employed in certain areas and accelerated SOC decomposition [16], therefore, decreasing input and increasing output of SOC impede the SOC accumulation.

### Vertical distribution of ΔSOCD

The SOC accumulation decreased significantly with soil depth. Smith et al. (2000) demonstrated that most changes in SOC occurred in the top 30 cm [70]. Roots are the most important source of soil carbon, and their proliferation plays a fundamental role in C cycling and SOC stabilization [71]. In a TG, 83% of the root biomass was distributed in the 0‒30 cm depth, whereas in an AG, 90% of which was distributed in this layer [44, 72]. The limited belowground biomass below a depth of 30 cm may cause a slight SOC increase in the AG+MG with deep soil horizons. The surface layer contained the most soil aggregates, and below this layer, the majority carbon accumulation occurred over a depth of 0‒30 cm. The SOC is transported from the surface layer to the subsurface layers and sequestered by humification, aggregation and translocation [32]. In a study conducted by An et al. (2009), the subsurface SOC did not increase significantly until 15 years after re-vegetation [47], which indicates that SOC restoration in the subsurface layer occurred later than in the surface layer. In one to two decades, the SOC accumulation over a depth of 0‒30 cm SOC accumulation may account for the total SOC accumulation. Over a longer time period, the SOC accumulation in deep soil layers should be calculated using linear regressions (Table 5). IPCC (2006) suggested that the influence of grassland management on SOC sequestration could be represented by the depth of 30 cm [73], the outcomes of our study, however, emphasized the importance of SOC changes in the subsurface below 30 cm for the national greenhouse gas inventories. The compacted soil surface in certain non-disturbed systems (e.g., AP and GE) tended to confine C additions to the upper few centimeters and resulted in C stratification because of limited incorporation of fresh organic residues in deeper layers [74]. That result may have accounted for the lower k value for AP and GE (Table 2). Vegetation cover with a deep root system is important because it provides a permanent input of organic matter into deep soil layers [75]. When greater SOC accumulation occurs in deeper horizons, which may be resistant to decomposition, C accumulation tends to be more stable.

### Factors regulating SOC processes

Numerous studies have revealed that climate conditions control changes in SOM content [13, 76, 77]. In our study, the surface layer SOC accumulation was related primarily to the RD and SOCD_pre. The accumulation of SOC can be determined by stand age [18]. Theoretically, SOC stocks should peak and level off after long-term conservative management; thus, the annual carbon sequestration rate may decrease to zero with an increase in RD. The grassland in the LAT<40° area was mainly AG with a relatively high SOC accumulation rate. Grasslands with a greater potential to increase soil C storage are those that have been depleted in the past by poor management [40, 78]. Studies on carbon sequestration potential are imperative, and the data supplemented by site sampling should be included to assess future carbon sequestration potential [9]. Accurate estimates of ΔSOCDs and their spatial distribution will highlight areas of high carbon sequestration potential prior to preservation and protection [79].

## Conclusions

The conservational managements of grasslands significantly increased the accumulation of surface layer SOC, and SOC accumulation decreased with an increase in soil depth. Although all management practices, EU, GE, AP and CA increased SOC accumulation, CA and EU resulted in lower carbon accumulation than did other practices. Based on the linear relationship of SOC accumulation between the surface and subsurface horizons, SOC changes in the subsurface horizon can be estimated from surface SOC measurements. SOC accumulation was correlated with RD, SOCD_pre, and the environmental factors, and varied considerably across different grasslands and management practices.

## Supporting Information

S1 Table (XLS)
