# Correction: Differential Effects of Conservational Management on SOC Accumulation in the Grasslands of China

**DOI:** 10.1371/journal.pone.0145590

**Published:** 2015-12-17

**Authors:** The PLOS ONE Staff

In the Funding section, the grant number from the funder National Basic Research Program of China is listed incorrectly. The correct grant number is: 2014CB954004. The publisher apologizes for the error.
